# Family interactions in toddlerhood influence social competence in preschool age: Accounting for genetic and prenatal influences

**DOI:** 10.3389/fpsyg.2022.975086

**Published:** 2022-11-28

**Authors:** Amanda M. Ramos, Elizabeth A. Shewark, David Reiss, Leslie D. Leve, Misaki N. Natsuaki, Daniel S. Shaw, Jody M. Ganiban, Jenae M. Neiderhiser

**Affiliations:** ^1^Department of Epidemiology, The University of North Carolina at Chapel Hill, Chapel Hill, NC, United States; ^2^Department of Psychology, Michigan State University, East Lansing, MI, United States; ^3^Child Study Center, Yale University, New Haven, CT, United States; ^4^Prevention Science Institute, University of Oregon, Eugene, OR, United States; ^5^Department of Psychology, University of California, Riverside, Riverside, CA, United States; ^6^Department of Psychology, University of Pittsburgh, Pittsburgh, PA, United States; ^7^Department of Psychological and Brain Sciences, George Washington University, Washington, DC, United States; ^8^Department of Psychology, The Pennsylvania State University, University Park, PA, United States

**Keywords:** genetic influences, evocative *r*GE, child social competence, prenatal influences, child regulation, parenting

## Abstract

Identification of early promotive and risk factors for social competence is important for fostering children’s successful social development; particularly given social competence is essential for children’s later academic and psychological well-being. While research suggests that the early parent–child relationship, genetics, and prenatal influences are associated with social competence, there is less research considering how these factors may operate together to shape children’s social competence in early childhood. Using a genetically informed sample from the Early Growth and Development Study (*N* = 561), we examined multiple levels of influence (i.e., genetic, prenatal, parenting, and child characteristics) on children’s social competence at 4.5 years old. Results from structural equation models showed adoptive mother overreactivity at 18 months was positively associated with child dysregulation at 27 months, which, in turn, was associated with lower levels of social competence at 4.5 years. Also, child reactivity at 18 months was independently associated with higher levels of adoptive mother overreactivity at 27 months, which, in turn, was associated with lower levels of social competence at 4.5 years. Finally, we found an evocative effect on adoptive fathers’ overreactivity at 18 months such that prenatal birth mother distress was negatively associated with adoptive fathers’ overreactivity at 18 months. Overall, this study found evidence for genetic influences, and bidirectional associations between parent and child in toddlerhood that are related to lower levels of social competence when children were 4.5 years old. We also found that the prenatal environment was associated with parenting, but not with child behavior directly. This study’s ability to simultaneously examine multiple domains of influence helps provide a more comprehensive picture of important mechanisms and developmental periods for children’s early social competence.

## Introduction

Children’s social competence, defined as children’s ability to engage in their social world appropriately (e.g., control their behaviors and emotions, display prosocial behaviors), are crucial skills to master for successful lifelong adjustment (Masten and Coatsworth, 1998; Blandon et al., 2010; Jones et al., 2015). Extensive research has documented the importance of early childhood, particularly early social interactions between parents and their children, for the development of social competence (Fabes et al., 2006; Karreman et al., 2006; Karam and Degnan, 2021). Extant literature has primarily focused on pinpointing parenting behaviors that promote and hinder the development of social competence (Masten and Coatsworth, 1998; Haskett and Willoughby, 2007; Driscoll and Pianta, 2011). However, even when there is evidence of parents’ effects on children’s social competence, the mechanisms that underlie these effects are ambiguous because most studies include parents and their biological children. This type of design makes it difficult to distinguish between environmentally-driven parent effects from effects that may be explained by genes shared by parents and their offspring, or prenatal effects (Knafo and Plomin, 2006; Moore and Neiderhiser, 2014). Additionally, while research has primarily focused on parenting effects, there is often evidence of bidirectional effects between children and their parents, pointing to the transactional nature of development (Bell, 1968; Kiff et al., 2011). Therefore, additional research is needed that considers how these factors (e.g., parenting, child characteristics, prenatal factors, and genetics) may operate individually or together to shape children’s social competence in early childhood. We employed a genetically informed design that would remove the confound of shared genes between child and parents to begin to address these gaps in the literature.

In early childhood, developmentally salient parenting behaviors like responsiveness and overreactivity (i.e., yelling, criticizing, and harsh punishment) are important promotive and risk factors, respectively, for the development of social competence (Masten and Coatsworth, 1998; Clark and Ladd, 2000; Haskett and Willoughby, 2007; Feldman and Masalha, 2010; Driscoll and Pianta, 2011). These different parenting behaviors are assumed to influence child development because they serve unique functions to socialize and model behaviors for the child. For example, parents’ responsivity to their child’s needs is critical for facilitating the development of a secure sense of self in the child and modeling emotionally competent behaviors (Thompson, 2000; Waters and Cummings, 2000). Longitudinal and concurrent studies have demonstrated the importance of parental responsivity for children’s development of social competence (Leerkes et al., 2009; Feldman and Masalha, 2010; Martin et al., 2010; Rispoli et al., 2013; Raby et al., 2015). One longitudinal study found that parental responsivity toward their infant was associated with higher levels of social competence in the preschool-aged child (Rispoli et al., 2013). On the other hand, overreactive and hostile parenting is detrimental to the child across multiple social outcomes, such as self-confidence, social competence, and overall well-being. Furthermore, children emulate the negative interaction styles with their parents with other individuals outside the home. Subsequently, these styles may adversely affect peer relationships, or children may have inhibitions about engaging in social behavior based on a history of harsh treatment by parents (Patterson, 1982; Anthony et al., 2005; Hartas, 2011). Concurrent and longitudinal studies have found that hostile parenting was associated with lower levels of social competence and fewer prosocial skills in preschool-aged children (Eddy et al., 2001; Laible et al., 2004; Anthony et al., 2005; Hartas, 2011; Walker and Mac Phee, 2011). Overall, this literature finds that responsive parenting and hostile/overreactive parenting shape children’s level of social competence.

While most previous research has focused on the impact of parenting on social competence, it is also likely that children play an active role in their development (Bell, 1968). This perspective has increasingly shifted attention to understand how parenting is influenced by individual children’s needs or demands and also incorporating transactional models of development (Patterson, 1982; Sameroff, 2009; Kiff et al., 2011). During early childhood, dysregulation and reactivity may be key early child behaviors that evoke behaviors from parents, and are also influenced by parents (Belsky, 1984; Kiff et al., 2011). Regulation is a multidimensional construct encompassing cognitive, behavioral, and emotional processes that allow the child to coordinate responses to environmental cues (Kopp, 1982; Grolnick and Farkas, 2002; McClelland and Cameron, 2012; Cole et al., 2019) and dysregulation is when an individual has impaired regulation, which this manuscript will focus on. In comparison, reactivity is characterized by the intensity and latency of one’s emotional and behavioral response to their environment and includes negative affect (Rothbart and Bates, 2006). Many studies examining the effects of reactivity and dysregulation reflect a broader conceptualization of these constructs, but this study will focus on specific aspects: anger proneness and behavioral dysregulation, measured by Attention-Deficit/Hyperactivity problems. Child anger proneness, in particular, is a salient child emotion that elicits negative parenting (Snyder et al., 2003; Kochanska et al., 2004; Paulussen-Hoogeboom et al., 2007; Shewark et al., 2021), and can also be detrimental to forming and maintaining peer relationships (Sirois et al., 2019). Behavioral dysregulation is associated with negative parenting (e.g., Morrell and Murray, 2003; Bridgett et al., 2009), decreases in positive parenting (e.g., Braungart-Rieker et al., 2001; Eisenberg et al., 2010), and later child maladjustment (e.g., higher externalizing; Eisenberg et al., 2010). Overall, higher levels of regulation and less negative affect are considered prerequisites for the healthy development of social competence because regulatory abilities help children to respond to their peers appropriately, and children who are overly angry have difficulty keeping good quality friendships (Rubin et al., 1995; Denham et al., 2003; Diener and Kim, 2004; Korja et al., 2017).

Research has also highlighted that early interactions between parenting behavior and child regulation and reactivity influence the development of social competence (Fabes et al., 2001; Eisenberg et al., 2010; Smith et al., 2014). For example, one study found that when parents used harsh parenting strategies in response to negative emotions (including anger) from their children, they had children who were more dysregulated and were less socially competent (Fabes et al., 2001). These negative interactions between parents and children are evidence of coercive family dynamics (Patterson, 1982; Scaramella and Leve, 2004; Smith et al., 2014). Coercion theory outlines a process between parent and child whereby parent hostility and child anger or noncompliance is mutually reinforced during interactions until an individual “wins” by one participant withdrawing or relenting (Patterson, 1982). While this coercive cycle is usually considered within interactions, these learned interactions can also be seen at a larger timescale (Van Ryzin and Dishion, 2012). This cycle can result in long-term destructive interaction patterns between the parent and child that negatively impact child social development (Scaramella and Leve, 2004; Smith et al., 2014). Therefore, parenting can influence children’s social competence by affecting children’s anger and regulatory abilities, but children’s anger and regulation could also impact social competence through eliciting parental responses associated with compromising children’s social competencies. Without accounting for child-driven effects, the parenting literature is unable to delineate whether the parenting behavior is the guiding force, or a response elicited by the child. To address these bidirectional processes occurring within the family, this study incorporates child-driven effects as a crucial factor that might be associated with early childhood social competence.

Last, even when bidirectional parent–child effects are identified within transactional models, the mechanisms underlying these effects are not fully understood. While a transactional model can help us determine potential directional patterns (i.e., parent vs. child-driven pathways), they are unable to clarify whether these relationships between parent and child are due to behavioral processes or shared genes. One way that genetics can influence parent–child relationships is through evocative gene–environment correlation (*r*GE). Specifically, how children are parented might be, in part, based on inherited characteristics of the child that elicit specific responses from the parent (Plomin et al., 1977; Scarr and McCartney, 1983; Knopik et al., 2017). Consistent with this possibility, studies have found that children’s early regulatory capacities, anger, and social competence are partially influenced by genetics (Edelbrock et al., 1995; Hudziak et al., 2003; Van Hulle et al., 2007; Roisman and Fraley, 2012; Van Ryzin et al., 2015). Furthermore, genetically informed studies have found that evocative *r*GE partially explains the relationship between child behaviors such as child regulatory behaviors and emotions and parenting in infancy and toddlerhood (Harold et al., 2013; Klahr et al., 2013, 2017; Natsuaki et al., 2013; Ulbricht et al., 2013; Klahr and Burt, 2014; Hajal et al., 2015). For example, Lipscomb et al. (2011) leveraged The Early Growth and Development Study (Leve et al., 2019) to remove the potential confound of genetic influences and found that child negative emotionality from infancy to toddlerhood was positively associated with higher levels of parent overreactivity from infancy to toddlerhood. Other studies, using this adoption sample and twin samples, have shown that evocative *r*GE effects on parenting in toddlerhood and preschoolers were associated with higher levels of disruptive and prosocial behaviors (Knafo and Plomin, 2006; Elam et al., 2014). For example, Elam et al. (2014) found that birth parent characteristics were associated with higher levels of parent hostility through child low social motivation. This result is evidence of evocative *r*GE because in an adoption design birth parents only provide genes and not the postnatal rearing environment; thus any influence the birth parent has on the child is assumed to be a genetic effect. These findings suggest that infancy and toddlerhood are important developmental periods to examine evocative *r*GE effects to help clarify how bidirectional parent–child processes might occur.

One final context to consider is the prenatal environment because the prenatal period is a particularly sensitive period for children’s early development, including social competence (Rice et al., 2007; Latimer et al., 2012; Behnke et al., 2013; Graignic-Philippe et al., 2014). The fetal programming hypothesis proposes that events that happen during the prenatal period can influence fetal development through reprogramming neural networks (Barker, 1998; Ping et al., 2015). For example, stress during pregnancy can increase hormone production that results in the altered functioning of the HPA axis, changes in glucocorticoid receptors, and alterations in neuroendocrine responses that are linked to fetal neural development (Matthews, 2002; Weinstock, 2005; Van den Bergh et al., 2020). Therefore, the prenatal environment might negatively alter the child’s neural network in the womb that starts the child at a higher threshold for being negative or dysregulated, which, in turn, has the potential to impact their normative developmental trajectory. This study focuses on prenatal distress (e.g., anxiety and depressive symptoms) because of its relevance to both early child reactivity (negative emotionality) and dysregulation (Feldman et al., 2009; Blair et al., 2011; Sharp et al., 2015; Korja et al., 2017), and social competence (Carter et al., 2001; O’Connor et al., 2002; Loomans et al., 2011; Dunkel Schetter and Tanner, 2012; Glover, 2014; Eichler et al., 2017). This hypothesis is supported by evidence that prenatal distress is associated with higher levels of negative affect and irritability in infants and toddlers (Davis et al., 2007; DiPietro et al., 2008; Glynn et al., 2018). Additionally, studies have also found that dysregulation or reactivity mediated the relationship between prenatal parent distress and later social development in children (Carter et al., 2001; DiPietro et al., 2008; Blair et al., 2011). In addition, based on previous findings of (1) the effects of prenatal distress on early child reactivity and regulation and (2) child-driven effects on parenting, child effects on parenting might also be indirectly influenced by the prenatal environment. This mediation effect is more challenging to test because of the inability of studies to separate prenatal and postnatal effects, resulting in biased estimates. Prenatal and postnatal stress are often correlated (i.e., if a mother is distressed prenatally, her parenting behaviors might also reflect that postnatally; Glover, 2015); therefore, different designs are needed distinguish between the prenatal and postnatal environment (Loehlin, 2016).

Within a family design where biological parents are raising their biological child(ren), researchers are unable to distinguish genetic, prenatal, and postnatal rearing environmental effects (e.g., parenting). Studies examining prenatal effects are unable to remove the bias of passive gene–environment correlation, where the genes of the mother might influence the prenatal environment (Knopik et al., 2017; Rice et al., 2018). For example, mothers with elevated depressive symptoms, which are heritable, might be more likely to experience depressive or anxiety symptoms during pregnancy (providing an environment correlated with their genetics), which could produce biased estimates in prenatal studies (i.e., larger prenatal effects). There are a few studies, including those examining the sample from the current report, that have examined prenatal effects within genetically informed designs (Rice et al., 2010; Kerr et al., 2013; Marceau et al., 2013; Neiderhiser et al., 2016; Gjerde et al., 2017; Hannigan et al., 2018; Liu et al., 2020). Previous studies have found prenatal effects (e.g., substance use, neonatal complications, prenatal risk) on toddler behaviors (Pemberton et al., 2010; Marceau et al., 2013; Liu et al., 2020); however, some found that the prenatal effect was at least partially due to genetic influences (Rice et al., 2007; Pemberton et al., 2010; Marceau et al., 2013; Hannigan et al., 2018; Liu et al., 2020). Finally, only a few studies have examined the influence of genetics, the prenatal environment, and the rearing environment on children’s social development in early childhood (Marceau et al., 2013, 2015; Neiderhiser et al., 2016; Liu et al., 2020). Therefore, the current study addresses the mechanistic pathways by which parent and child bidirectional processes unfold—parent-driven, child-driven, genetically influenced, or prenatally influenced—to influence children’s early social competence.

**Figure 1:**
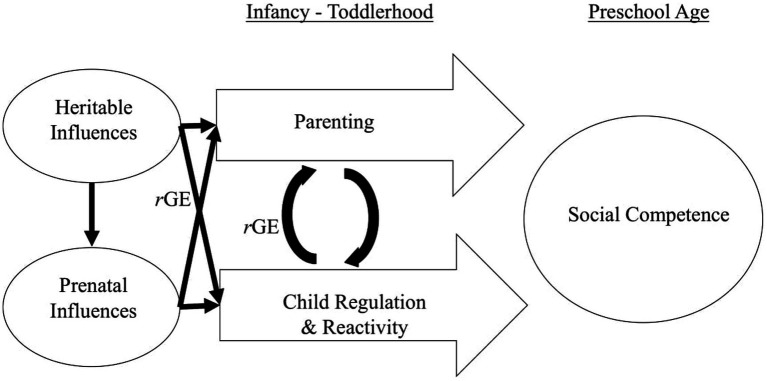
Conceptual model of genetic, prenatal, and the bidirectional process between parent and child on social competence in early childhood.

Specifically, we address this gap by employing a parent-offspring adoption design to simultaneously examine the effects of genetic factors, prenatal distress, child behaviors (i.e., dysregulation and anger proneness), and parenting (i.e., responsivity and overreactivity) on child social competence at preschool age (Figure 1). The parent-offspring adoption design allows us to better distinguish genetic, prenatal environmental influences, and postnatal rearing environmental influences as birth parents provide only their genes (and for the birth mother, the prenatal environment), but not the rearing environment, and the adoptive parents provide only the rearing environment because they are genetically unrelated to the child. In this study, genetic influences are indicated by birth parent agreeableness and emotion dysregulation. These are reasonable birth parent proxies (which are included to estimate genetic influences) for children’s social competence, early anger proneness, and dysregulation because they are both moderately heritable (Bouchard Jr. and McGue, 2003; Tackett et al., 2013), and both incorporate aspects of regulation and emotion. While the adoption design does not definitively distinguish prenatal influences from genetic influences, the inclusion of both birth mother and birth father characteristics provides some leverage for making this distinction as the birth father does not provide the prenatal environment (Loehlin, 2016). To advance our understanding of the complex nature of transactional family processes, this study examined both positive and negative parenting behaviors (responsivity and overreactivity). Finally, we included both mothers and fathers based on the importance of both parents in the development of children (Karam and Degnan, 2021).

We hypothesized that (1) there would be a bidirectional process between child dysregulation and reactivity and parenting during toddlerhood that, in turn, would be associated with child social competence at age 4.5 years. We hypothesized that child dysregulation would be associated with lower levels of parent responsiveness and higher levels of coercive parenting in the toddler period, leading to lower levels of social competence in children at preschool age. (2) We also hypothesized that we would find evidence of evocative *r*GE, such that birth parent (BP) temperament would be associated with parenting through children’s early reactivity and dysregulation, and (3) these evocative pathways would be associated with child social competence at 4.5 years old. (4) Lastly, we hypothesized that prenatal distress would be negatively associated with child social competence through child reactivity and dysregulation.

## Materials and methods

### Participants

Participants were from the Early Growth and Development Study (EGDS), a longitudinal adoption design, with 561 linked sets of adopted children, adoptive parents, and birth parents (Leve et al., 2019). Recruitment of these families occurred through 45 adoption agencies in 15 states in the USA, and families were eligible to participate if the adoption was domestic, placement occurred within 3 months (*M* = 5.57 days, *SD* = 11.30 days), the child was placed with a nonrelative, the child had no major medical conditions, and the birth parents and adoptive parents could understand English at an 8th grade level. The adopted children were majority male (57.3%), and about half White (54.5%), with 17.8% multiethnic, 13.2% African American, and 13.4% Hispanic, 0.5% America Indian or Alaskan Native, 0.2% Asian, and 0.2% Native Hawaiian or Pacific Islander, and 0.2% unknown or not reported. Demographic information about the adoptive parents (APs) and birth parents (BPs) are provided in Table 1. BPs were generally younger than APs, had less education, and lower income.

**Table 1:** Full Sample descriptives.

	Adoptive parent 1 (*N* = 561)	Adoptive parent 2 (*N* = 552)	Birth mother (*N* = 556)	Birth father (*N* = 210)
Age at child birth [*M (SD)*]	37.43 (5.59)	38.30 (5.83)	24.35 (6.03)	26.08 (7.77)
**Race/ethnicity**				
White	91.8%	90.4%	70.1%	69.9%
African American	3.9%	4.9%	13.3%	11.5%
Hispanic	2%	1.6%	6.7%	9.6%
More than 1 race	0.4%	1.1%	4.9%	4.8%
Asian	0.9%	0.5%	1.8%	0%
American Indian or Alaskan Native	0.2%	0%	2.5%	0.5%
Native Hawaiian or Pacific Islander	0%	0.5%	0.2%	0.5%
Other	0.4%	0.9%	0.5%	3.3%
Income (median)	$100,000–$125,000		<$15,000	$15,000–$40,000
Education (median)	College degree		High school degree	

### Measures

#### BP temperament

We used latent temperament factors that were constructed for both birth mothers (BMs) and birth fathers (BFs) to estimate genetic influences previously created (Shewark et al., 2021). We used the following factors: emotion dysregulation and agreeableness. Emotion dysregulation consisted of attentional control, activation control, fear, and frustration subscales from the Adult Temperament Questionnaire—short form completed when the child was 18 months old (ATQ; Rothbart et al., 2000). Agreeableness consisted of the sociability subscale from the ATQ, and the nurturance and intimate relationship subscales from the Harter Adult Self-Perception Profile completed when the child was three to 6 months old (Messer and Harter, 1986). Higher scores on the emotion dysregulation factor are indicative of higher levels of dysregulation with lower scores indicative of higher levels of attentional control and activation. Higher agreeableness indicates higher levels of sociability and better interpersonal relationships. Birth fathers were less likely to participate, most often because they could not be located, which resulted in between ~60%–71% missing data. Missing data were handled with Full Information Maximum Likelihood (Graham, 2003). More information on the construction of these factors is provided in Shewark et al. (2021). In analyses, BM and BF factor scores were used and paths were constrained to be equal to provide a single genetic influence estimate for each temperament construct. The inclusion of BF data within the genetic estimates provides a better representation of the full genetic influence and can help distinguish this from the prenatal effects.

#### Birth mother prenatal distress

When the child was approximately 5 months old, BMs reported their depressive and anxiety symptoms during pregnancy. To aid mothers in their recall, interviewers helped mothers generate a list of life events that occurred throughout the pregnancy to create a Life History Calendar (Freedman et al., 1988; Caspi et al., 1996). BMs completed shortened versions of the Beck Anxiety Inventory (BAI; Beck and Steer, 1993) and the Beck Depression Inventory (BDI; Beck and Steer, 1993), adapted by the study team for the pregnancy period. Mothers who endorsed sadness or anhedonia for at least a 2-week period during pregnancy were asked to rate an additional five items from the BDI. Similarly, mothers were asked about anxiety symptoms with an additional set of four items from the BAI. Both subscales were on a 4-point Likert scale and had good reliability (BDI α = 0.86; BAI α = 0.80). Example items include: “I have been able to laugh and see the funny side of things” and “Things have been getting on top of me.” Prenatal depressive and anxiety symptoms were combined by summing their scores (*r* = 0.55, *p* < 0.001).

#### Adopted child dysregulation

To capture dysregulation, we used adoptive parent reports on the Attention-Deficit/Hyperactivity Problems subscale of the Child Behavior Checklist (Achenbach et al., 1987). This subscale includes 6 items on a scale from 1 (Not True) to 3 (Very True), for example, “Cannot concentrate.” This subscale shows good reliability at 18 months [adoptive mother (AM) α =0.85; adoptive father (AF) α =0.87] and 27 months (AM α =0.77; AF α =0.80). AM and AF scores were averaged to create composites (18 months, inter-rater *r* = 0.39; 27 months: *r* = 0.42).

#### Adopted child anger proneness

To capture anger proneness, we used parent reports on the anger proneness subscale of the Toddler Behavior Assessment Questionnaire (Goldsmith, 1996). This subscale consists of 28 items that assess the child’s likelihood of presenting anger in situations, for example, “When you did not allow your child to do something for her/himself, for example, dressing or getting into the car seat, how often did your child try to push you away?” Each parent reported on these child behaviors at 18 (AM α =0.89; AF α =0.89) and 27 months (AM α =0.85; AF α =0.87). AM and AF scores were averaged to create composites (18 months: *r* = 0.42; 27 months: *r* = 0.44).

#### Adoptive parents’ parenting

We assessed both positive and negative parenting behaviors at 18 and 27 months. For *positive parenting behaviors*, we assessed parents’ responsivity. To assess parents’ responsivity using the Home Observation for Measurement of the Environment (HOME) Inventory. The HOME was designed to measure the emotional and social responsiveness of the parent to the child. This assessment was completed by the study interviewers upon the completion of the in-home interview. The responsivity subscale consists of 11 items. An example item is: “mother/father responds to the child’s vocalizations with a verbal or vocal response.” This measure showed good reliability for both parents at both waves (18 months: AM α = 0.76, AF α = 0.71; 27 months: AM α = 0.58, AF α = 0.70). As responsivity was negatively skewed, we performed a reciprocal transformation of it with the intention of keeping the high scores meaning higher responsivity.

For *negative parenting behaviors,* adoptive parents self-reported on the Parenting Scale (Arnold et al., 1993) at 18 and 27 months for which we used the overreactivity subscale, which reflects displays of anger, meanness, and irritability. An example item is: “When my child misbehaves, I get so frustrated or angry that my child can see that I am upset.” This subscale consisted of 10 items on a 7-point Likert scale and showed good reliability for both parents across both timepoints (18 months: AM α = 0.79, AF α = 0.77; 27 months: AM α = 0.79, AF α = 0.77).

#### Adopted child social competence

We used parent reports on child social competence at child age 4.5 years on the Social Skills Rating System (Gresham and Elliott, 1990), using the total social skills score that includes 39 items (AM α =0.87; AF α =0.87) that includes items about cooperation, communication, responsibility, and self-control during interactions with peers. Example items include “Ends disagreements with you calmly” and “Requests permission before leaving the house.” Parents respond to items on a 3-point Likert scale. AM and AF scores were averaged to create a composite (*r* = 0.48, *p* < 0.001).

#### Covariates

Openness of adoption, child sex, obstetric complications (e.g., prenatal complications, neonatal complications, substance use), parent age at the child’s birth, education, and income were tested as covariates on all study variables. Significantly related covariates were controlled for in subsequent analyses by regressing them out of the study constructs and creating standardized z-scores.

#### Analytic strategy

Hypotheses were tested using structural equation models in Mplus 8 (Muthén and Muthén, 1998–2012). Due to power concerns, we had to model the birth parent indices and child behaviors in separate models, resulting in four models: BP emotion dysregulation-adopted child dysregulation, BP emotion dysregulation-adopted child anger proneness, BP agreeableness-adopted child dysregulation, and BP agreeableness-adopted child anger proneness. There was a small amount of missing data across ages (18 months: 3%, 27 months: 5%, 4.5 years: 18.36%), so we used full information maximum likelihood (FIML) estimation to reduce bias of missing data (Graham, 2003). Fit statistics were used to examine the fit of the models including, Chi-square goodness of fit index (*p* > 0.05), CFI (0.90 or above), SRMR (less than 0.08), and RMSEA (less than 0.08). Main effects and indirect effects were examined within each model. Birth mother and birth father emotion dysregulation and agreeableness paths were constrained to be equal in the models to estimate a single genetic influence and help separate the genetic and prenatal influence.

## Results

Descriptive statistics and correlations among the raw study variables are presented in Tables 1, 2, respectively. Correlations reflect that adoptive parents’ overreactivity was significantly associated with early child regulation, reactivity, and social competence with moderate effect sizes (*r*’s range from −0.25 to 0.30). Also, child early dysregulation and reactivity were significantly associated with later social competence (*r*’s range from −0.19 to −0.28). Finally, birth parent temperament was significantly correlated with social competence at 4.5 years (*r* = −0.12 to 0.12).

**Table 2:** Correlations and mean and standard deviations of the raw study variables.

	1	2	3	4	5	6	7	8	9	10	11	12	13	14	15	16
1. BP emotion dysregulation	--	−0.67^**^	0.11^*^	−0.02	0.05	0.03	−0.02	0.01	−0.04	−0.01	0.03	−0.01	−0.08	0.07	−0.01	−0.12^*^
2. BP agreeableness	−0.47^**^	--	−0.05	−0.02	−0.10^*^	−0.02	0.02	−0.01	0.04	0.01	−0.04	−0.07	0.02	−0.06	−0.01	0.08
3. Prenatal distress	0.25^**^	−0.21^**^	--													
4. AM overreactivity, 18 months	−0.01	−0.03	−0.05	--												
5. AF overreactivity, 18 months	−0.03	−0.07	−0.10^*^	0.34^**^	--											
6. AM responsivity, 18 months	0.03	−0.01	0.01	−0.02	−0.01	--										
7. AF responsivity, 18 months	0.01	0.04	−0.07	−0.01	0.01	0.54^**^	--									
8. AC dysregulation, 18 months	0.00	−0.08	−0.02	0.22^**^	0.12^**^	0.05	0.08	--								
9. AC Anger proneness, 18 months	−0.03	−0.02	−0.09^+^	0.24^**^	0.24^**^	0.02	0.03	0.36^**^	--							
10.AM overreactivity, 27 months	−0.04	0.06	−0.07	0.73^**^	0.27^**^	0.02	0.03	0.24^**^	0.26^**^	--						
11.AF overreactivity, 27 months	−0.02	−0.03	−0.11^**^	0.27^**^	0.69^**^	−0.05	−0.05	0.16^**^	0.24^**^	0.27^**^	--					
12. AM responsivity, 27 months	0.01	−0.02	0.00	−0.07	−0.06	0.23^**^	0.22^**^	0.10^*^	−0.05	0.02	−0.02	--				
13. AF responsivity, 27 months	0.01	0.04	−0.07	−0.04	−0.04	0.10^**^	0.27^**^	0.10^*^	−0.01	−0.01	0.00	0.37^**^	--			
14. AC dysregulation, 27 months	0.04	−0.08	−0.01	0.27^**^	0.10^**^	0.02	0.10^+^	0.67^**^	0.26^**^	0.27^**^	0.24^**^	0.04	0.07	--		
15. AC anger proneness, 27 months	0.00	−0.08	−0.04	0.23^**^	0.24^**^	0.05	0.05	0.35^**^	0.68^**^	0.30^**^	0.25^**^	−0.02	−0.01	0.37^**^	--	
16. AC social competence 4.5 years	−0.07	0.12^*^	0.00	−0.23^**^	−0.18^**^	0.03	0.02	−0.19^**^	−0.27^**^	−0.25^**^	−0.12^*^	0.14^**^	0.02	−0.27^**^	−0.28^**^	
*Mean*	0.00	0.00	8.70	1.86	1.90	10.58	10.05	5.13	3.40	2.07	2.06	10.70	10.27	4.59	3.58	48.87
*Standard deviations*	0.83	0.77	7.43	0.60	0.60	1.14	1.57	2.03	0.62	0.62	0.62	0.78	1.43	2.13	0.60	7.90

BP - Birth mother correlations are shown down the columns, Birth father correlations are shown across the rows.

+ *p* < 0.06;

* *p* < 0.05;

** *p* < 0.001.

### Birth parent emotion dysregulation models

**Figure 2:**
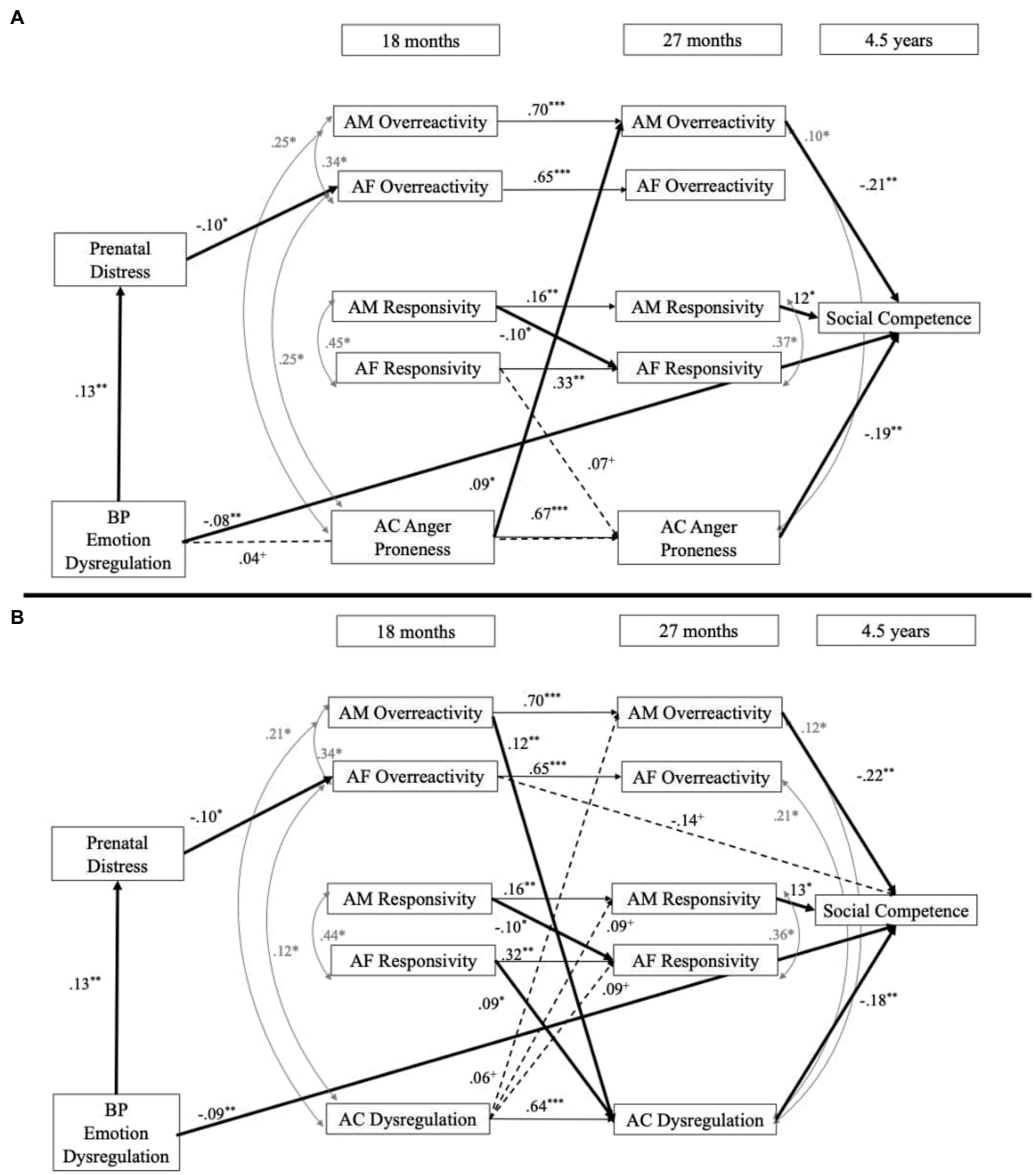
Birth parent emotion dysregulation models. **(A)** Presents the results with adopted child reactivity and **(B)** presents the results with adopted child dysregulation. BP, birth parent; AM, adoptive mother; AF, adoptive father; AC, adopted child. Nonsignificant paths are not included to assist with readability but were included in the statistical model. Solid lines indicate a significant effect and dashed lines indicate a trend-level association. ^+^*p* < 0.07, ^*^p < 0.05, ^**^p < 0.01, ^***^p < 0.001. Indirect prenatal effect BP emotion dysregulation-Prenatal distress-AF overreactivity: β = −0.01, *SE* = 0.01, *p* = 0.06. Panel **A** indirect effect AM overereactivity-AC dysregulation-Social competence: β = −0.02, *SE* = 0.01, *p* = 0.05. Panel **B** indirect effect AC reactivity-AM overreactivity-Social competence: β = −0.02, *SE* = 0.01, *p* = 0.05.

Both models fit well: child dysregulation [*χ*^2^(25) = 29.82, *p* = 0.23, RMSEA = 0.02, CFI = 1.00, SRMR = 0.02], and child reactivity [*χ*^2^(26) = 29.15, *p* = 0.30, RMSEA = 0.02, CFI = 1.00, SRMR = 0.02]. Results (depicted in Figure 2) indicate that BP emotion dysregulation was negatively associated with child social competence at 4.5 years. In addition, adoptive mothers’ overreactivity was negatively and adoptive mothers’ responsivity was positively associated with child social competence, but adoptive fathers’ parenting was not associated with child social competence. Adopted child dysregulation was also negatively associated with child social competence. Finally, we found that birth parent emotion dysregulation was positively associated with birth mother’s prenatal distress and that prenatal distress was negatively associated with adoptive fathers’ AF overreactivity at 18 months.

We found differences when examining the bidirectional effects between child dysregulation and child reactivity in relation to parenting. For the *child dysregulation model* (Figure 2A), we found that adoptive mothers’ overreactivity at 18 months was positively associated with child dysregulation at 27 months; however, adoptive fathers’ overreactivity was not associated with child’s dysregulation. Additionally, adoptive fathers’ responsivity was positively associated with child dysregulation at 27 months, but not adoptive mothers’ responsivity. We also found that adoptive mothers’ responsivity at 18 months was negatively associated with adoptive fathers’ responsivity at 27 months. Additionally, child dysregulation at 18 months was not associated with adoptive parents’ parenting at 27 months. For the *child reactivity model* (Figure 2B), we found that adoptive parents’ parenting at 18 months was not associated with children’s reactivity at 27 months. However, we found that child reactivity at 18 months was positively associated with adoptive mothers’ overreactivity at 27 months; however, child reactivity was not associated with other parenting behaviors. We also found that adoptive mothers’ responsivity at 18 months was negatively associated with adoptive fathers’ responsivity at 27 months.

#### Indirect effects

There was an indirect effect from adoptive mothers’ overreactivity at 18 months to child social competence at 4.5 years through adoptive mothers’ overreactivity at 27 months (β = −0.15, *SE = 0*.05, *p* < 0.01). There was a marginally significant indirect effect from birth mother emotion dysregulation to adoptive fathers’ overreactivity through prenatal distress (β = −0.01, *SE = 0*.01, *p* = 0.06). For the *child dysregulation model,* adoptive mothers’ overreactivity at 18 months to child social competence at 4.5 years was significant through child dysregulation at 27 months (β = −0.02, *SE = 0*.01, *p* = 0.05). Additionally, there was an indirect effect of child dysregulation at 18 months to child social competence through child dysregulation at 27 months (β = −0.11, *SE = 0*.04, *p* < 0.05). Finally, adoptive fathers’ responsivity to child social competence was not significant through child dysregulation at 27 months (β = 0.01, *SE = 0*.01, *p* > 0.05). There were no additional significant evocative *r*GE pathways in this model. For the *child reactivity model,* child reactivity at 18 months was associated with child social competence at 4.5 years through adoptive mother overreactivity at 27 months (β = −0.02, *SE = 0*.01, *p* = 0.05). However, because birth parent emotion dysregulation was not associated with adopted child reactivity, there were no additional significant evocative *r*GE pathways.

### Birth parent agreeableness models

**Figure 3:**
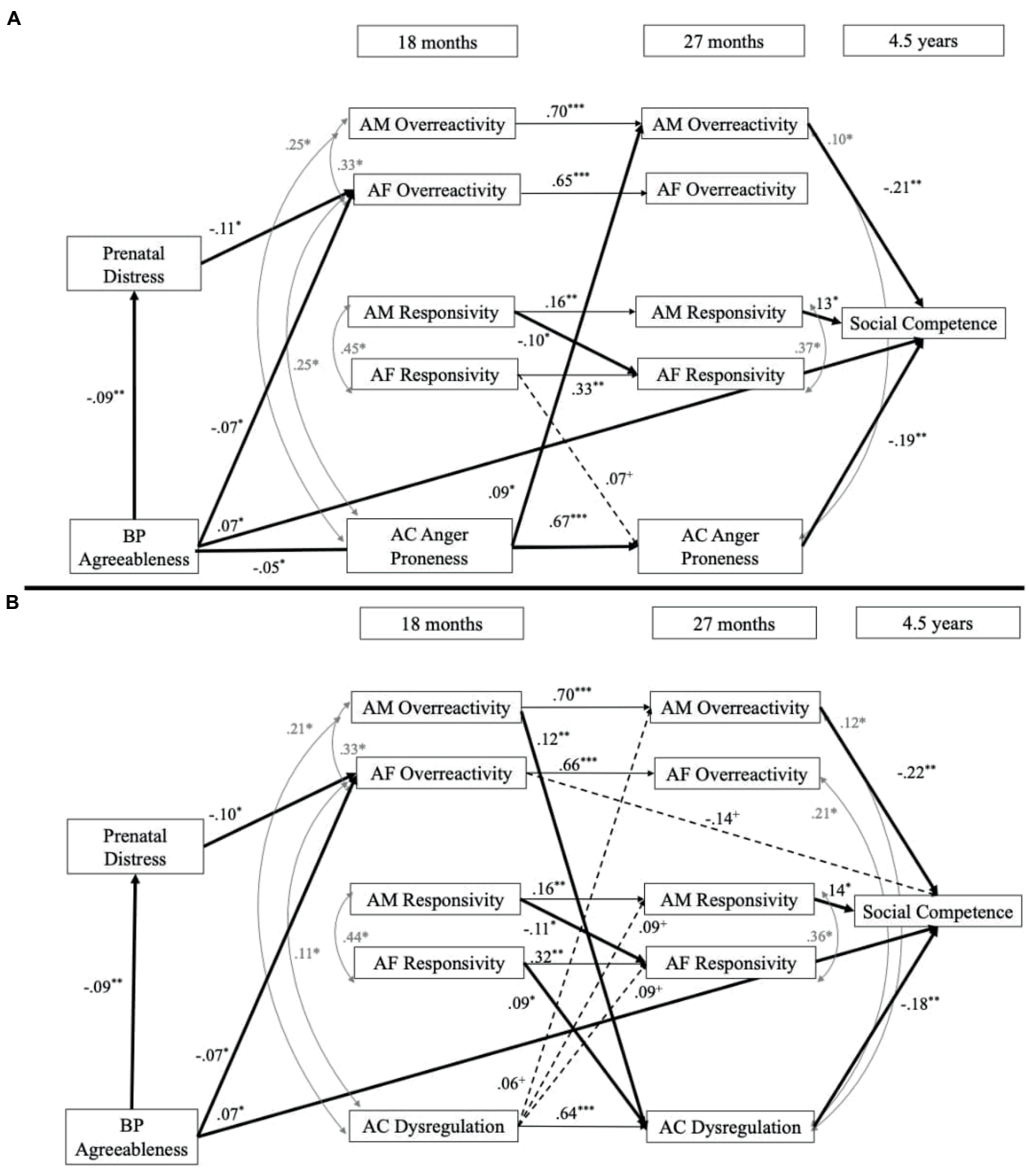
Birth parent agreeableness models. **(A)** Presents the results with adopted child reactivity and **(B)** presents the results with adopted child dysregulation. BP, birth parent; AM, adoptive mother; AF, adoptive father; AC, adopted child. Nonsignificant paths are not included to assist with readability but were included in the statistical model. Solid lines indicate a significant effect and dashed lines indicate a trend-level association. ^+^*p* < 0.07, ^*^p < 0.05, ^**^p < 0.01, ^***^p < 0.001. Indirect prenatal effect BP emotion dysregulation-Prenatal distress-AF overreactivity: β = 0.01, *SE* = 0.01, *p* = 0.06. Panel **A** indirect effect AM overereactivity-AC dysregulation-Social competence: β = −0.02, *SE* = 0.01, *p* = 0.05. Panel **B** indirect effect AC reactivity-AM overreactivity-Social competence: β = −0.02, *SE* = 0.01, *p* = 0.05.

Both of the models fit well: child dysregulation [*χ*^2^ (25) = 26.34, *p* = 0.39, RMSEA = 0.01, CFI = 1.00, SRMR = 0.04] and child reactivity [*χ*^2^ (26) = 23.51, *p* = 0.60, RMSEA = 0.00, CFI = 1.00, SRMR = 0.02]. Results (depicted in Figure 3) showed that birth parent agreeableness was positively associated with child social competence at 4.5 years old. Birth parent agreeableness was also negatively associated with child reactivity at 27 months, but not child dysregulation. Birth parent agreeableness was also negatively associated with prenatal distress and adoptive fathers’ overreactivity at 18 months. We also found that birth mother prenatal distress was negatively associated with adoptive fathers’ overreactivity at 18 months. In addition, both adoptive mothers’ overreactivity and responsivity were associated with child social competence (β = −0.22, *p* < 0.05; β = 0.13, *p* < 0.05, respectively), but adoptive fathers’ parenting behaviors were not associated with child social competence. Child dysregulation during toddlerhood was negatively associated with child social competence.

When examining the bidirectional effects of parenting and child behavior, we found for the *child dysregulation model* (Figure 3A) that adoptive mothers’ overreactivity at 18 months was positively associated with child dysregulation at 27 months; however, adoptive fathers’ overreactivity was not associated with child dysregulation. Additionally, adoptive fathers’ responsivity was positively associated with child dysregulation at 27 months, but not adoptive mothers’ responsivity. We also found that adoptive mothers’ responsivity at 18 months was negatively associated with adoptive fathers’ responsivity at 27 months. Additionally, child dysregulation at 18 months was not associated with adoptive parents’ parenting at 27 months. For the *child reactivity model* (Figure 3B), we found that adoptive parents’ parenting at 18 months was not associated with child reactivity at 27 months. However, we found that child reactivity at 18 months was positively associated with adoptive mothers’ overreactivity at 27 months; however, child reactivity was not associated with other parenting behaviors. We also found that adoptive mothers’ responsivity at 18 months was negatively associated with adoptive fathers’ responsivity at 27 months.

#### Indirect effects

There was an indirect effect from adoptive mothers’ overreactivity at 18 months to child social competence at 4.5 years through adopted mothers’ overreactivity at 27 months (β = −0.15, *SE = 0*.05, *p* < 0.01). The indirect effect from birth parent emotion dysregulation to adoptive fathers’ overreactivity through prenatal distress also was marginally significant (β = 0.01, *SE = 0*.01, *p* = 0.06). For the *child dysregulation model,* adoptive mothers’ overreactivity at 18 months was associated with child social competence through child dysregulation at 27 months (β = −0.02, *SE = 0*.01, *p* = 0.05). Additionally, there was an indirect effect of child dysregulation at 18 months to child social competence through child dysregulation at 27 months (β = −0.11, *SE = 0*.04, *p* < 0.05). Finally, adoptive fathers’ responsivity to child social competence was not significant through child dysregulation at 27 months (β = 0.01, *SE = 0*.01, *p* > 0.05). For the *child reactivity model,* child reactivity was associated with child social competence at 4.5 years through adoptive mothers’ overreactivity at 27 months (β = −0.02, *SE = 0*.01, *p* = 0.05). However, there were no additional significant evocative *r*GE pathways.

## Discussion

This study is one of the few genetically informed projects to examine genetic, prenatal, and postnatal rearing influences in early childhood and specifically in relation to children’s early social competence. The findings indicate different bidirectional pathways by which parents and children can influence each other and negatively influence children’s social competence. Additionally, there was evidence of unique genetic influences on social competence, evidence of evocative *r*GE, as well as an association between prenatal distress and fathers’ overreactivity. Together these findings provide a more comprehensive picture of the longitudinal effects of risk factors across multiple domains for children’s social skill development.

First, we found direct associations of genetic influences (birth parent emotion dysregulation and birth parent agreeableness), child early regulatory capacities (dysregulation and reactivity), and adoptive mother parenting behaviors, but not adoptive father parenting on children’s social competence at 4.5 years old. The current study extends previous work on children’s social competence in this sample (Van Ryzin et al., 2015), which examined gene–environment interaction and found that genetic influences buffered children’s social competence at 6 years old against less sensitive parenting. We considered another genetic mechanism (evocative *r*GE) by examining bidirectional processes between parent and child across early childhood, as well as directly examining prenatal effects on social competence in preschool-age children. Our study’s findings support previous research that social competence is subject to genetic influence (DiLalla et al., 2012; Van Ryzin et al., 2015; Battaglia et al., 2017), and that early regulatory capacities promote the development of social competence (Hubbard and Coie, 1994; Cole et al., 2004; Denham, 2006).

In addition, our work supports previous findings that adoptive mothers’ parenting behaviors can be both deleterious and promotive to children’s early social competence (Masten and Coatsworth, 1998; Clark and Ladd, 2000; Haskett and Willoughby, 2007; Driscoll and Pianta, 2011). However, fathers’ parenting was not uniquely associated with children’s social competence above and beyond adoptive mothers’ parenting. This pattern of results for mothers’ effects is supported by the majority of studies using only mothers (Laible et al., 2004; Leerkes et al., 2009; Rispoli et al., 2013; Raby et al., 2015), which might be due to mothers most often being the primary caregiver. However, in a study of both parents, Martin et al. (2010) found that having at least one parent who uses positive parenting behaviors is enough to help promote the child’s social competence, and then the child could hit a “ceiling”—the child is scoring at the highest level of the scale—and the other parent provides no added benefit. The current study’s finding does not negate the importance of the father and his role in children’s development as we only considered two parenting behaviors and fathers may help to advance the child’s social competence development in different ways. For example, some research suggests that fathers use more activation parenting (e.g., intense play with limit setting) that is focused on challenging the child while setting boundaries (Feldman and Shaw, 2021; Karam and Degnan, 2021). Therefore, examining these more traditional, and to some extent, more maternal-centric parenting behaviors (i.e., responsiveness) might not capture the independent role that fathers play in promoting or undermining children’s social competence. These direct effects highlight the complex nature of equifinality, whereby multiple risk and promotive factors might come together over time to influence social competence in early childhood.

Second, consistent with our hypothesis, we found bidirectional pathways between parent and child that were associated with lower levels of child social competence at 4.5 years, while accounting for both genetic and prenatal influences. Specifically, we found both a child-driven pathway (i.e., child anger proneness at 18 months was associated with lower levels of social competence at 4.5 years through adoptive mother overreactivity at 27 months) and a parent-driven pathway (i.e., adoptive mother overreactivity at 18 months was associated with less social competence through child dysregulation at 27 months). These indirect pathways support previous non-genetically informed work exploring the unfolding of a coercive cycle between children and parents (Patterson, 1982), but provide new insights into its implications for social competence during early childhood (Shaw and Bell, 1993; Eddy et al., 2001; Smith et al., 2014). More specifically, these findings along with prior work (Eisenberg et al., 1996, 2010; Fabes et al., 2001) provide evidence that these coercive cycles between mother and child could be detrimental to children’s early social competence. These early negative transactions are associated with decreased children’s social skills prior to school entry which could set the child up for more negative child behaviors at school-age (Shaw et al., 1994, 1998; Smith et al., 2014). These indirect pathways could also suggest a more involved process whereby the coercive cycle between mother and child might increase the child’s dysregulation, which, in turn, could negatively impact their social competence; however, this study was unable to test this directional association. The findings that both early emerging child-and parent-directed coercive cycles are associated with deficits in social competence highlight how early negative parent–child relationships can decrease critical social milestones that place a child at risk for later maladjustment.

We also found developmentally salient bidirectional pathways that show directional associations and might suggest how child and parent responses mutually reinforce each other on a longer timescale. Specifically, we found that child anger proneness at 18 months was associated with adoptive mother overreactivity at 27 months, while adoptive mother overreactivity and adoptive father responsiveness at 18 months were associated with child dysregulation at 27 months. Child anger proneness eliciting negative parenting is consistent with prior literature on child reactivity (Bridgett et al., 2009; Liu et al., 2020), but expands upon this work by also considering parenting pathways to child anger proneness. In contrast, when examining child dysregulation, we found only parent to child pathways. Specifically, we found that the child’s dysregulation is being influenced by negative parenting, consistent with prior work (Grolnick and Farkas, 2002; Karreman et al., 2006). Finally, the finding that fathers’ responsivity at 18 months was associated with more child dysregulation at 27 months was unexpected. While studies generally find that being a responsive parent is important for the child’s development (Davidov and Grusec, 2006; Feldman et al., 2009; Rispoli et al., 2013; Raby et al., 2015), a meta-analysis found that responsiveness was not associated with child regulation (Karreman et al., 2006). A further consideration is that fathers’ concept of responsiveness might serve different functions dependent on the child behavior (Kochanska and Aksan, 2004). This idea is supported by research finding that when fathers interact with their children, their synchrony of play is categorized by high peaks of intensity (e.g., overstimulation) without limit setting that could increase the child’s dysregulation (Feldman, 2003; Paquette, 2004; Meuwissen and Carlson, 2018; Karam and Degnan, 2021). Overall, these findings allude to the potential differences these child behaviors might have within a dyad. Generally, child reactivity is an emotional response to the environment (i.e., parent) that poses a challenge to the parent, who, in turn, responds to the child (Paulussen-Hoogeboom et al., 2007). Meanwhile, regulation is developed over time by the child and supported by the parents who help the child to regulate their emotions and behaviors at an early age. While parents’ overreactivity is in response to the child’s displays of anger, parents’ overreactivity could be also an indicator of dysregulated behavior. Thus, parent overreactivity could be modeled to the child, which disrupts and over time hinders the child’s growing regulatory abilities.

This study also was able to distinguish between genetic and environmental influences to examine whether bidirectional effects between parent and child might be partially due to genetic influences. Although we did not find support for our main hypotheses regarding evocative *r*GE—whereby birth parent temperament would be associated with parenting at 27 months through child reactivity or dysregulation, we did find an evocative *r*GE effect on adoptive fathers’ overreactivity at 18 months from birth parent agreeableness. However, this study did not model the specific child behavior that was evoking the fathers’ behavior. One possible earlier child behavior could be children’s early sociability or positive affect—which is heritable (Van Hulle et al., 2007), as birth parent agreeableness is indicative of higher sociability and better interpersonal relationships. Therefore, even before toddlerhood, children might be eliciting behaviors from their parents, partially based on their genetically influenced characteristics. This result supports a meta-analysis finding that evocative *rGE* has a larger effect in early childhood and decreases over time (Klahr and Burt, 2014). This pattern also supports previous genetically informed studies that found evocative effects on fathers and not mothers (Ulbricht et al., 2013; Hajal et al., 2015), which could suggest that specific parenting behaviors from fathers might be more susceptible to characteristics of the child at an early age that are at least partially due to genetic influences. We did not, however, find other evocative effects on mother or father responsivity, suggesting that these particular genetic propensities (emotion dysregulation, agreeableness) might be relevant only for overreactivity in infancy, whereas other genetic propensities (e.g., psychopathology) might be more relevant for other parenting behaviors (Trentacosta et al., 2019).

Although we did not find support for our hypothesis with either direct associations between prenatal distress and child behaviors (anger proneness, dysregulation, social competence) or an indirect pathway, we found that higher birth mother prenatal distress was associated with less adoptive fathers’ overreactivity at 18 months. This finding could partially support the influence of prenatal distress on children’s early behavior, as prenatal distress is presumably associated with father overreactivity through a child behavior that was not measured. Some research suggests that prenatal distress blunts children’s cortisol responses, which is associated with less reactivity, and this could be why fathers respond less negatively to their children (Laurent et al., 2013; O’Connor et al., 2013). This study did include anger proneness at 18 months, but found no indirect pathway from prenatal distress to overreactivity at 27 months; however, future work may explore earlier child reactivity or other behaviors in infancy that might be impacted by maternal prenatal distress. We also found that both birth parent emotion dysregulation and birth parent agreeableness were associated with birth mothers’ prenatal distress; however, the indirect effects to adoptive father overreactivity were not significant. Thus, the genetic influences of the parents were correlated with the prenatal environment (which might be indicative of passive *r*GE), but the indirect effect to fathers’ parenting was not significant. Therefore, the prenatal association is likely to be an environmental influence on parenting, supporting previous work on the importance of prenatal distress (Booth et al., 1991; Barker et al., 2011).

### Limitations

The results of the current study should be considered within the context of a few limitations. The first limitation is our ability to fully disentangle genetic and prenatal influences. This issue is challenging because birth mothers are reporting on both their behaviors and their prenatal experiences, increasing the potential for reporter bias. However, compared to other study designs, the adoption design is better able to disentangle genetic and prenatal influences, especially if birth fathers participate and are modeled with birth mothers (Loehlin, 2016). The inclusion of birth fathers’ information in this study allows us to model the full genetic influence (birth mother and birth father), and better separate the genetic and prenatal influences. However, our birth fathers had lower levels of participation and might have influenced our estimates of genetic influences. Birth father missing data were handled with Full Information Maximum Likelihood (FIML) to account for the missing data and would likely introduce minimal bias in the birth father estimates. FIML has been found to be a superior approach to handling large amounts of missing data compared to listwise deletion and substitution methods and performs equally well when compared to multiple imputation (Graham, 2003; Lang and Little, 2018).

Second, adoptive parents are reporting on both parenting behavior (overreactivity and responsivity) and child behaviors; therefore, increasing the potential for reporter bias. To partially minimize this bias in our study, we combined parent reports of the child behaviors so that one parent was not providing the report on themselves and their child alone. Parent report of child behaviors can be influenced by the parents’ own personality and child behavior (e.g., Treutler and Epkins, 2003), thus future studies should examine if consistent patterns of effects emerge with observational measures. It should be noted that observational measures provide a snapshot in time of behaviors that might not always have ecological validity depending on the observational setting (Gardner, 2000). Studies of child social competence suggest that there is small to moderate convergence of parent report and observation (Kotler and McMahon, 2002; Hawes and Dadds, 2006; Bennetts et al., 2016), suggesting that parents and observations can provide unique insights into child behavior. Third, these findings might not be generalizable to other populations, as the experiences of adoptive parents can be different from other parents (e.g., at-risk families, non-adoptive families). Additionally, our adoptive parents were majority White and highly economically advantaged. However, one study suggests that the potential for the restricted range of behaviors (i.e., less negative behaviors) does not differentially impact child outcomes compared to non-adoptive families (McGue et al., 2007). Finally, the prenatal experiences of birth mothers placing their children for adoption might be more unique than other samples (e.g., substance use and emotional difficulties). However, these mothers’ experiences are not largely different from those of other mothers who did not place their child for adoption (Marceau et al., 2016; Ramos et al., 2020). They typically have similar levels of substance use, but often have a higher prevalence of depressive episodes that decrease over time, compared to national averages.

In addition, we were not able to consider trimester specific effects of prenatal distress on child social competence given the constraints of secondary data analysis; therefore, the findings reflect prenatal distress across pregnancy. Future studies should consider the effects of trimester specific prenatal distress within a genetically informed study. Finally, while there is value in understanding specific aspects of reactivity and dysregulation given the potential for unique mechanistic pathways and effects (e.g., McClelland and Cameron, 2012; Shewark et al., 2021), this specificity might limit our findings to these specific aspects of reactivity and dysregulation. Therefore, future work should continue to consider the implications of both specific aspects of reactivity and dysregulation on child development as well as broader conceptualizations of reactivity and dysregulation that can capture the complex nature of these constructs. There has been work done in early and later childhood to capture the multidimensional nature of dysregulation (e.g., Bellani et al., 2012; McClelland and Cameron, 2012), and future work can consider the utility of this measure alongside other multidimensional measures of regulation in infancy and toddlerhood to improve measurement of this critical construct.

### Conclusion

Despite these caveats, this study is one of a few to examine multiple levels of influence (genetic, prenatal, child behaviors, and parenting) on the development of early social competence. The current study simultaneously assessed multiple levels of influence to better capture the complex multifaceted mechanisms of influence on children’s social competence. These findings suggest not only that child and parent interactions are impacting child social competence, but that some of the parenting behaviors might be elicited by children’s behaviors and influenced by both genetic and prenatal influences. Identifying the presence of coercive cycles in early childhood within a genetically informed design also provides converging evidence for the importance of the bidirectional processes between parent and child. Additionally, the lack of finding direct prenatal influences on child behavior does not imply that these influences do not exist. Rather, the child behaviors examined in this report may not be the most sensitive to the effects of prenatal distress. Future genetically informed studies should continue to examine the influence of the prenatal environment as a risk mechanism on positive child outcomes. For example, child cortisol could be examined as a mechanism through which the prenatal environment influences child development. The genetic influences found here are not trivial and suggest important mechanisms in the development of social competence. Therefore, future studies should continue using genetically informed designs to examine the bidirectional relationship between parents and children that influence children’s social competence. Ultimately, examining the influence of these environment and genetic factors simultaneously within a genetically informed design can help researchers examine important mechanisms and important developmental periods where change might be more influential.

## Data availability statement

Data are not publicly available due to privacy or ethical restrictions, but are available on request from the corresponding author.

## Ethics statement

The studies involving human participants were reviewed and approved by the University of Oregon Institutional Review Board (Project No.: 08082016.007; Title: The Early Growth and Development Study Pediatric Cohort). Written informed consent to participate in this study was provided by the participants’ legal guardian/next of kin.

## Author contributions

AR and JN developed the study idea. AR conducted the analyses, with assistance from ES, and drafted the manuscript. DR, DS, JG, LL, MN, and JN designed the larger study and supervised data collection. DR, DS, JG, LL, MN, ES, and JN provided conceptual and written feedback on the manuscript. All authors contributed to the article and approved the submitted version.

## Funding

This project was supported by R01 HD042608 from NICHD and NIDA (PI Years 1–5: DR; PI Years 6–10: LL), as well as OBSSR and NIH, R01 DA020585 from NIDA, NIMH, OBSSR, and NIH. Research was also supported by the Office of The Director, National Institutes of Health under Award Number UH3OD023389. AR was also supported in part by a training grant from the Institute of Education Sciences (R305B090007) and NIEHS F32ES031832. ES was supported by NICHD F32HD098780.

## Conflict of interest

The authors declare that the research was conducted in the absence of any commercial or financial relationships that could be construed as a potential conflict of interest.

## Publisher’s note

All claims expressed in this article are solely those of the authors and do not necessarily represent those of their affiliated organizations, or those of the publisher, the editors and the reviewers. Any product that may be evaluated in this article, or claim that may be made by its manufacturer, is not guaranteed or endorsed by the publisher.
